# Effect of Ganduqing on common cold: A protocol for systematic review and meta-analysis based on existing evidence

**DOI:** 10.1097/MD.0000000000021678

**Published:** 2020-08-14

**Authors:** Xiaomin Wang, Tingting Liao, Yongcan Wu, Demei Huang, Caixia Pei, Zhenxing Wang, Fei Wang

**Affiliations:** Hospital of Chengdu University of Traditional Chinese Medicine, Sichuan, China.

**Keywords:** Ganduqing, common cold, systematic review, study protocol

## Abstract

**Background:**

The common cold is an infectious viral disease of the upper respiratory tract that has become the most frequent infectious disease in humans. Currently there is no cure for the common cold, and treatment typically focuses on alleviating symptoms. Although antiviral treatment is an important focus of current research, more than 200 viral strains have been associated with the common cold, making antiviral drug interventions difficult. Ganduqing is a Chinese medicinal preparation composed of Astragalus and Shegan. Several randomized controlled trials have been conducted to evaluate treatment of the common cold, but their effectiveness and safety have not been scientifically evaluated. In this study, we will systematically examine the efficacy and safety of Ganduqing in patients with common cold.

**Methods:**

The following electronic databases will be systematically and comprehensively searched: Cochrane Library, EMBASE, PubMed, Science Network, China National Knowledge Infrastructure, China Biomedical Literature Database, Wanfang Database and Chinese Journal Database, for randomized controlled trials that used Ganduqing for treating the common cold through June 2020. The primary outcomes were signs and symptoms of the common cold, including cough, sore throat, fever, nasal congestion and runny nose. Secondary outcomes included changes in the percentage of neutrophils and lymphocytes, and recurrence. Study selection, data extraction and quality assessment will be independently conducted by 2 researchers. Meta-analyses incorporating data derived from the literature will conduct using Review Manager (RevMan) v.5.3 and Stata 14 software. The Grading of Recommendations, Assessment, Development and Evaluations framework will be used to assess the quality of evidence derived from the meta-analyses.

**Results:**

This systematic review and meta-analysis aims to provide an evidence of Ganduqing for the common cold and will be disseminated through publications in peer-reviewed journals and/or presentations at scientific conferences.

**Conclusions:**

This systematic review will provide evidence for the efficacy and safety of Ganduqing in treating common colds.

**Trial registration number:**

INPLASY202060073

## 1 Introduction

The common cold is a viral infection of the upper respiratory tract in humans. Although more than 200 virus strains have been implicated in the common cold, rhinovirus infections are the most common.^[1]^ Primary symptoms include sore throat, rhinitis, runny nose, fever, and nasal congestion.^[2]^ The incidence of the common cold tends to decrease with age. Children normally experience ∼5 colds per year, while adults average 2 to 3 colds per year, and the elderly will normally have ∼1 illness per year.^[1,3]^ Although the common cold is self-limiting, severe symptoms will limit the productivity of affected patients.^[4]^ Direct economic losses caused by the common cold in the United States are estimated to be as high as $17 billion each year.^[5]^

Treatments for the common cold are designed to alleviate the patient's clinical symptoms. The first generation of antihistamines may effectively relieve common symptoms such as sneezing and runny nose, but sedative antihistamines have common side effects and are restricted in children.^[6]^ Intranasal or oral decongestants may effectively relieve nasal congestion, but are limited by adverse effects such as drug rhinitis and recurrence of congestion.^[7]^ Non-steroidal anti-inflammatory drugs may effectively relieve fever and sore throat caused by the common cold, and may have beneficial effects on cough.^[8]^ Although cough medicines are widely prescribed by clinicians, research has shown that the efficacy of cough medicines for treating the common cold is poor.^[9]^ It is worth noting that antiviral drugs have received attention in recent years; however, since the common cold could be caused by numerous viruses, it has been virtually impossible to develop uniform antiviral drugs in clinical practice. Many antiviral drugs have been effective in vitro, but no satisfactory result was produced from clinical trials.^[10]^

Complementary and alternative medicine treatments have been the focus of a growing body of research in recent years. Echinacea has been widely used for common cold in Europe and North America, but a recent meta-analysis concluded that Echinacea products have no obvious benefit. In addition, COLD-fX (CVT-E002), an extract from the roots of North American ginseng (Panax quinquefolius), has been reported to reduce the relative risk and duration of respiratory symptoms by ∼50%; however, the quality of the research on CVT-E002 remains questionable and the actual efficacy is unknown.^[11]^ Therefore, it is meaningful to develop new drugs to manage common cold.

Ganduqing (Astragalus, Shegan) has shown positive antiviral effects in animal models exposed to influenza virus that have been related to endogenous Interferon α and induced Interferon β, as well as inhibition of viral activation.^[12]^ In patients with the common cold who took Ganduqing, cytokine Human β-defensin-2 levels increased, which may be an important mechanism for treatment.^[13]^ In recent years, several randomized controlled trials (RCTs) using Ganduqing to treat the common cold have been conducted; therefore, we aim to initiate a systematic review and meta-analysis to evaluate the effectiveness and safety of Ganduqing in the management of common cold.

## 2 Methods

### 2.1 Protocol register

The research protocol is developed in accordance with recommendations of the Systematic Review and Meta-Analysis Protocols,^[14]^ which was intended to guide the development of protocols for systematic reviews and meta-analyses that evaluate therapeutic efficacy. This study has been registered on International Platform of Registered Systematic Review and Meta-analysis Protocols and has been assigned a registration number, International Platform of Registered Systematic Review and Meta-analysis Protocols 202060073.

### 2.2 Ethics

All eligible studies were approved by the local Institutional Review Board and Ethics Committee. All participants were required to provide written informed consent; therefore, no further ethical approval was required.

### 2.3 Eligibility criteria

The Population, Intervention, Comparison and Outcomes (PICO) principles will be applied to the research design. The inclusion criteria are as follows:

(1) Article was written in Chinese or English;
(2) A randomized controlled trial;
(3) Conformed to expert consensus regarding the standard diagnosis and treatment of common colds^[15]^; and
(4) Conformed to the traditional Chinese medicine (TCM) definition of qi deficiency syndrome.

The exclusion criteria are as follows:

(1) Use of a non-RCT design;
(2) Use of a quasi-RCT study design;
(3) Important data were unavailable;
(4) Repeated publication;
(5) Animal model experiments;
(6) Reviews or case reports; and
(7) Interventions that included other TCM therapies such as acupuncture or massage.

### 2.4 Types of intervention

Ganduqing or modified Ganduqing was administered to the treatment group. The control group was given Lianhua Qingwen capsules or Compound Paracetamol capsules. Patients were enrolled in RCTs within 3 days of disease onset. Patients were excluded if the RCTs included any form of external treatment from TCM. No restriction is placed on medication dosage or course of treatment.

### 2.5 Outcome measures

#### 2.5.1 Primary outcome indicator

(1) Total effective rate of clinical treatment;
(2) Improvement of cough, sore throat, runny nose, fever, and nasal congestion; and
(3) Changes (%) in neutrophil and lymphocyte levels during treatment.

#### 2.5.2 Secondary outcome indicator

The effective number of patients who relapsed with the common cold is a secondary outcome variable. The prognostic indicators of this study include worsening of illness, hospital admission or death. In addition, security measures, adverse events and costs will be considered.

### 2.6 Literature search strategy

Xiaomin Wang and Tingting Liao will conduct an extensive search for relevant literature published prior to June 2020 in eight major databases. The search strategies were using the following key words and search terms: #1 Common Cold; #2 Colds, Common; #3 Common Colds; #4 Cold, Common; #5 Coryza, Acute; #6 Acute Coryza; #7 Catarrh; #8 Catarrhs; #9 Ganduqing; # 10 Modified Ganduqing; #11 #1 or #2 or #3 or #4 or #5 or #6 or #7 or #8; #12 #9 or #10; #13 #11 and #12.

### 2.7 Study selection and data extraction

**Figure 1:**
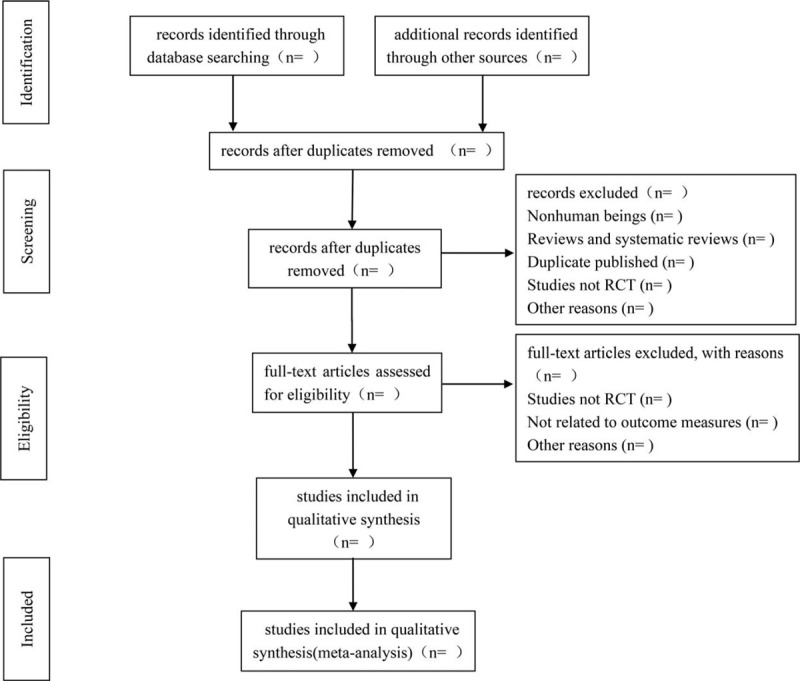
Flow diagram of the study selection process. RCT = randomized controlled trial.

Two researchers (Xiaomin Wang, Tingting Liao) will independently search, evaluate, and extract data from the literature. The 2 researchers will compare results with each other according to established standards. Discrepancies will be resolved by a third reviewer (Zhenxing Wang). The research selection process was summarized in a Preferred Reporting Items for Systematic Reviews and Meta-Analyses flowchart (Fig. 1). All research articles will be managed by EndNote X9. Data will be extracted from the literature following a pre-established document extraction table that included:

(1) Title, author, source, and publication date of the article;
(2) Basic characteristics (sample size, gender, age, diagnostic criteria, intervention measures, duration of intervention, and outcomes and adverse events); and
(3) Methods (research design, randomization methods, blinding methods, allocation concealment and other problems).

If necessary, the original author(s) of target article will be contacted to obtain additional information. If the data were completed or unavailable, the relevant research was discarded.

### 2.8 Risk of bias assessment

Two researchers (Yongcan Wu, Caixia Pei) will independently evaluate each study based on the Cochrane Collaboration's tool for assessing the risk of bias,^[16]^ which contained random sequence generation, allocation concealment, blinding of participants and personnel, blind assessment of results, incomplete results data, selective reports and other biases. Each project was classified as low bias risk, high bias risk or unclear bias risk. Inconsistencies between the researchers will be resolved through discussion. Persistent differences will be adjudicated by a third researcher (Zhenxing Wang).

### 2.9 Data synthesis

RevMan 5.3 will be used for statistical analysis of the selected literature. The results of continuous variables will be calculated as the standard mean differences (SMDs) and 95% confidence interval (CI), while categorical data will be expressed as risk ratios (RRs). Initially, the results will be compared on the same scale using a fixed-effect model to determine the *P*-value and *I*^2^-value to assess the level of heterogeneity between trials. Based on the results of the heterogeneity tests, we will decide whether to use fixed effect models or random effect models (specifically: if (*I*^2^ > 50% or *P* < .1, random effect models will be used, otherwise fixed effect models will be used). If heterogeneity (*I*^2^ > 50% or *P* < .1) is detected, the source of the heterogeneity will be explored. Subgroup analysis will be used to evaluate different interventions and other factors in the control group. If we still cannot find the source of heterogeneity, a narrative and qualitative summary will be provided. Stata 14 software will be used to analyze the sensitivity of the results. In addition, we will use a funnel plot to assess the reporting bias if ten or more studies are included.

### 2.10 Quality of evidence

The quality of evidence for each selected study will be evaluated by using the Grading of Recommendations Assessment Development and Evaluation system. The following 5 items will be investigated: research limitations, inconsistencies, indirect evidence, accuracy and publication bias. The quality of evidence will be divided into 4 levels: high, moderate, low or very low.

### 2.11 Ethics and dissemination

This systematic review and meta-analysis will not require ethical approval since there are no data used in our study that are linked to individual patient data. We aim to publish this systematic review in a peer-reviewed journal and present the results at an international scientific conference.

## 3 Discussion

Clinical trials examining TCM interventions for the common cold have significantly increased in recent years. More and more Chinese medicine programs are being developed for preventing and treating the common cold; however, no satisfactory results have been obtained. Ganduqing (containing Astragalus and Shegan) has shown clinical benefits in a variety of animal models and clinical trials, including fever reduction, resolving external symptoms, benefiting qi and detoxification at low cost. A single clinical trial found that Ganduqing can significantly improve clinical symptoms of patients with colds, such as cough and fever,^[17]^ and some clinical trials have shown that Ganduqing can resolve discomfort of the throat in ordinary cold patients.^[18]^ Despite these positive findings, no study has systematically evaluated the role of Ganduqing in treating the common cold. At present, clinical trials have only examined its effect on the recurrence of few common cold. In 1 study, Ganduqing significantly reduced the recurrence rate of the common cold over a 3-month period compared with the control group.^[19]^ In summary, the purpose of this study is to evaluate the effect of Ganduqing on the common cold.

### 3.1 Amendments

If any amendments are necessary in this protocol, we will provide the date of each amendment, describe the specific change(s) and provide the reason for each change.

## Acknowledgment

We would like to thank TopEdit (www.topeditsci.com) for English language editing of this manuscript.

## Author contributions

**Conceptualization:** Xiaomin Wang, Tingting Liao.

**Data curation:** Xiaomin Wang, Tingting Liao.

**Formal analysis:** Xiaomin Wang, Demei Huang.

**Investigation:** Caixia Pei, Yongcan Wu.

**Methodology:** Xiaomin Wang, Zhenxing Wang, Yongcan Wu.

**Resources:** Xiaomin Wang, Yongcan Wu.

**Software:** Zhenxing Wang, Caixia Pei.

**Supervision:** Fei Wang.

**Writing – original draft:** Xiaomin Wang, Tingting Liao.

**Writing – review & editing:** Fei Wang, Zhenxing Wang.
